# How to Kill the Honey Bee Larva: Genomic Potential and Virulence Mechanisms of *Paenibacillus larvae*

**DOI:** 10.1371/journal.pone.0090914

**Published:** 2014-03-05

**Authors:** Marvin Djukic, Elzbieta Brzuszkiewicz, Anne Fünfhaus, Jörn Voss, Kathleen Gollnow, Lena Poppinga, Heiko Liesegang, Eva Garcia-Gonzalez, Elke Genersch, Rolf Daniel

**Affiliations:** 1 Department of Genomic and Applied Microbiology and Göttingen Genomics Laboratory, Institute of Microbiology and Genetics, Georg-August-University Göttingen, Göttingen, Germany; 2 Department for Molecular Microbiology and Bee Diseases, Institute for Bee Research, Hohen Neuendorf, Germany; Charité-University Medicine Berlin, Germany

## Abstract

*Paenibacillus larvae*, a Gram positive bacterial pathogen, causes American Foulbrood (AFB), which is the most serious infectious disease of honey bees. In order to investigate the genomic potential of *P. larvae*, two strains belonging to two different genotypes were sequenced and used for comparative genome analysis. The complete genome sequence of *P. larvae* strain DSM 25430 (genotype ERIC II) consisted of 4,056,006 bp and harbored 3,928 predicted protein-encoding genes. The draft genome sequence of *P. larvae* strain DSM 25719 (genotype ERIC I) comprised 4,579,589 bp and contained 4,868 protein-encoding genes. Both strains harbored a 9.7 kb plasmid and encoded a large number of virulence-associated proteins such as toxins and collagenases. In addition, genes encoding large multimodular enzymes producing nonribosomally peptides or polyketides were identified. In the genome of strain DSM 25719 seven toxin associated loci were identified and analyzed. Five of them encoded putatively functional toxins. The genome of strain DSM 25430 harbored several toxin loci that showed similarity to corresponding loci in the genome of strain DSM 25719, but were non-functional due to point mutations or disruption by transposases. Although both strains cause AFB, significant differences between the genomes were observed including genome size, number and composition of transposases, insertion elements, predicted phage regions, and strain-specific island-like regions. Transposases, integrases and recombinases are important drivers for genome plasticity. A total of 390 and 273 mobile elements were found in strain DSM 25430 and strain DSM 25719, respectively. Comparative genomics of both strains revealed acquisition of virulence factors by horizontal gene transfer and provided insights into evolution and pathogenicity.

## Introduction

Honey bees (*Apis mellifera*) are among the most important livestock due to their role in pollination of many crops, fruits, and wild flowers [1]. Nowadays, 90% of commercial pollination is performed by managed honey bees and the demand for this service is growing faster than the global stock of honey bees [2], [3]. This might lead to an imbalance of supply and demand in the near future. Therefore, honey bee health is of crucial importance not only for apiculture but also for agriculture and human food security.

Honey bees are attacked by numerous pathogens and parasites including viruses, bacteria, fungi, and metazoans [4]. *Paenibacillus larvae* is one of the two bacterial species known to be pathogenic for honey bees. This Gram-positive, spore-forming and peritrichously flagellated bacterium is the causative agent of American Foulbrood (AFB) [5], a fatal, globally spread epizootic disease. Although AFB only kills infected honey bee larvae, it eventually leads to the collapse of entire colonies when left untreated. AFB is also considered very contagious; therefore, it is a notifiable disease in most countries.

The spores of *P. larvae* are the infectious form. Larvae are most susceptible to infection during the first 36 hours after egg hatching when a few spores per larva are sufficient to initiate infection; at later larval developmental stages spore doses needed to successfully infect a larva are too high to occur under natural conditions [6], [7]. Soon after ingestion, spores germinate in the larval midgut, where they massively proliferate for several days without destroying the integrity of the midgut epithelium [8]. At a later stage of infection, *P. larvae* breaches the peritrophic matrix [9] and the epithelial barrier and invades the haemocoel. Recent studies revealed that *P. larvae* destroys cell-cell and cell-matrix junctional structures to follow the paracellular route from the gut lumen into larval tissue. Breaching of the epithelium was shown to coincide with larval death [8].

The species *P. larvae* comprises four different genotypes named ERIC I to ERIC IV [5], [10]. All four genotypes differ in several phenotypic characteristics [5], [11], [12], most importantly in virulence [7], [13]. Epidemiological studies showed that only ERIC I and II are frequently isolated from AFB-diseased colonies [14]–[17]. Thus, ERIC I and II are the most important genotypes with respect to infection of honey bee larvae. The genotype-specific differences in virulence between *P. larvae* ERIC I and II correspond to the time it takes to kill infected larvae [5], [7]. Members of ERIC II are rather fast killers with an LT_100_ of approximately seven days while members of ERIC I are killing more slowly (LT_100_ approximately 12 days) [18]. These differences in virulence on the individual larval level also influence the virulence on the colony level [14].

Our knowledge on *P. larvae* and the pathogenesis of AFB increased tremendously over the past decade [19]. Two draft genome sequences of two *P. larvae* strains are available [20], [21], but with large numbers of remaining gaps. In addition, several putative virulence factor genes are differentially present in the genomes of the four ERIC-genotypes of *P. larvae* [5]. However, most molecular aspects of this important pathogen still remain elusive.

Here, we present the whole genome sequences of *P. larvae* genotypes ERIC I (strain DSM 25719) and ERIC II (strain DSM 25430) and a comparative analysis to elucidate both, the general pathogenic mechanisms of *P. larvae* and the genotypic differences in virulence. The study is focused on the identification of potential virulence genes in each genome and analysis of genotype-specific differences between the two *P. larvae* genotypes ERIC I and ERIC II.

## Results and Discussion

### General Genomic Features

**Figure 1:**
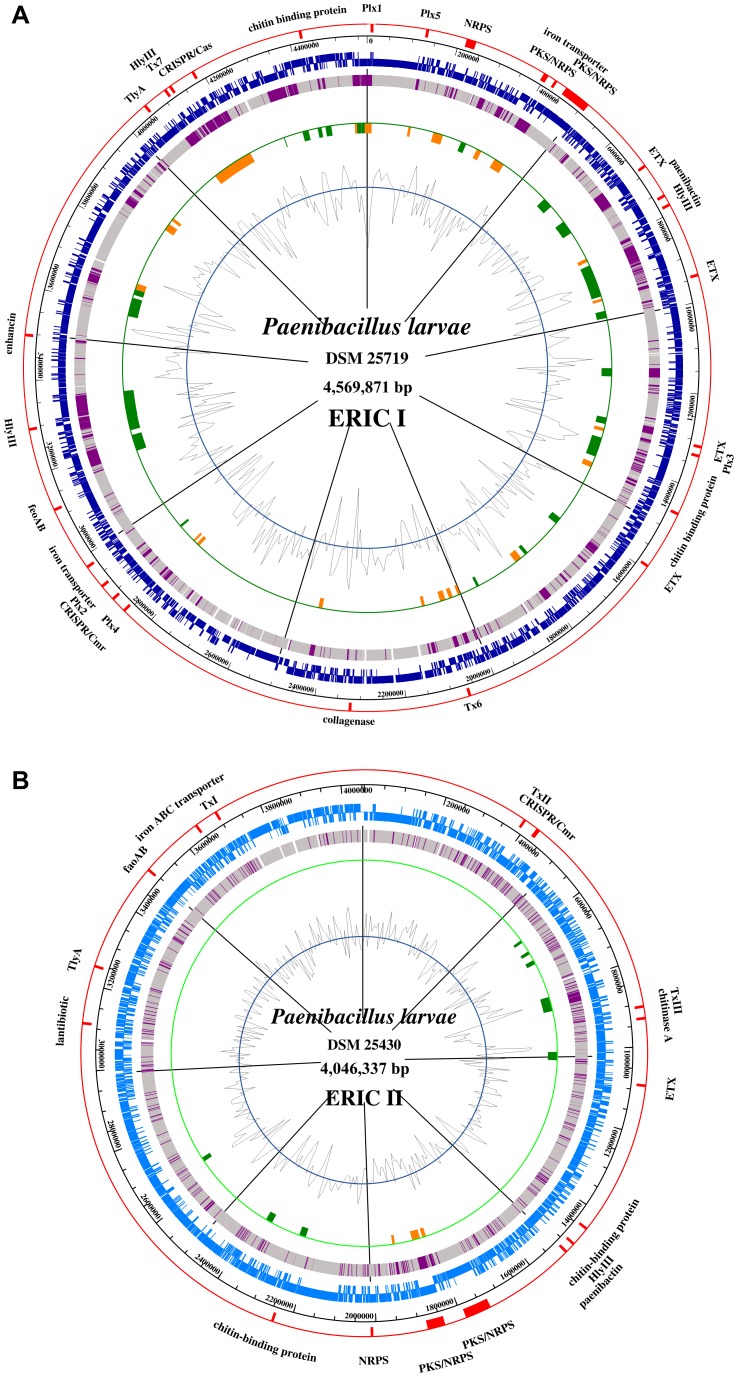
Maps of the *P. larvae* DSM 25719 (A) and DSM 25430 (B) chromosome The different circles represent (from inside): (a), GC content; (b), strain-specific regions (orange) and prophages (green); (c), genes present in both analyzed strains (grey) and genes found only in one strain (purple); (d), all ORFs clockwise and anticlockwise or (blue); (e), scale; (g), highlighted genes, gene clusters, including toxins, potential virulence factors mentioned in the manuscript (red).

We have sequenced, manually curated and annotated the genomes of two *P. larvae* isolates representing the two genotypes ERIC I (strain DSM 25719) and ERIC II (strain DSM 25430). The general features of both genomes are presented in Table 1. The complete genome of strain DSM 25719 consisted of 4,579,589 bp whereas the one of strain DSM 25430 harbored 4,056,006 bp. The number of replicons was identical in both strains. A total of 4,868 and 3,928 protein-encoding genes were predicted for DSM 25719 and DSM 25430, respectively. The higher overall genome size of strain DSM 25719 is mainly due to the presence of additional prophage regions (Table 1). We could identify 8 putatively phage-related regions within the DSM 25430 genome. However, all of them appeared incomplete. Within the DSM 25719 genome 22 phage-related regions have been identified. (Figure 1, Table 1) [22].

**Table 1:** General genomic features of the *P. larvae* strains.

Feature	DSM 25719 (ERIC I)	DSM 25430 (ERIC II)
Status	8 contigs	2 contigs
Chromosome size	4,569,871 bp (7 contigs)	4,046,337 bp (closed)
Plasmid size	9,718 bp (closed)	9,669 bp (closed)
GC content	44%	45%
No. rRNA genes	23	25
No. tRNA genes	79	81
No. ORFs	4,868	3,928
No. pseudogenes	75	99
No. transposases	256	366
No. phage integrases and site-specific recombinases	17	24
No. phage regions	22	8
Phage regions size	693.4 kbp	153.4 kbp

IS elements are transposable DNA fragments that provide the structural basis for rearrangements of genomic fragments, incorporation of foreign DNA into the genome, and homologous recombination [23], [24]. We found 390 mobile elements (transposases, integrases and recombinases) in the genome of DSM 25430, and 273 in that of DSM 25719 (Table 1). A striking difference of both strains is the high copy number of mutator-type transposases in the DSM 25430 genome. Nevertheless, the large number of mobile genetic elements and prophage regions in the genomes of both *P. larvae* strains suggested frequent genome rearrangements and a high degree of genome plasticity.

This feature of the *P. larvae* genome has also been recognized recently, when Chan and co-workers tried an update and draft annotation of the *P. larvae* sequence [21] originally published by Qin and co-workers [20]. The number of contigs could be reduced from 646 [20] to 388 [21] but still no complete genome sequence could be obtained. The annotation even based on the original 646 contig-version. The authors hypothesized that the fragmentation of their assembly may be due to long genomic repeats that could not be bridged by their sequencing strategy. Our data confirms the existence of genomic regions containing repeats and repetitive sequences, which indeed were difficult but not impossible to sequence. We were able to close the sequence of the DSM 25430 replicons, which are now available without any gap. The final sequence of the DSM 25719 strain consists of only seven contigs with length 3,663,994 bp, 771,602 bp, 86,545 bp, 77,837 bp, 12,832 bp, 8,981 bp and 8,080 bp.

In both strains we found a 9.7 kb-circular plasmid designated pPLA1_10 in DSM 25719 and pPLA2_10 in DSM 25430 with almost identical sequences differing in only 49 bases (Figure S1). A gene encoding a putative replication initiation factor (REP) was identified in both replicons. A plasmid of similar size (pPll9.4) has been reported for ERIC II-strains [12]. The existence of a plasmid in strains of *P. larvae* ERIC I was contradictory to an earlier report in which pPll9.4 was found exclusively in ERIC II-strains and, therefore, had been considered as characteristic for ERIC II [12]. Screening an international collection of 65 ERIC I strains and 30 ERIC II strains revealed that indeed no other strains of the ERIC I genotype harbored the pPLA1_10 plasmid indicating that the one found in DSM 25719 was strain-specific but not genotype-specific.

### Metabolism

#### Energy metabolism

*P. larvae* is a facultative anaerobic organism that grows preferentially under aerobic conditions. In addition to typical oxygen-dependent respiration, both strains are able to utilize nitrate as alternative electron acceptor. Genes encoding a putative respiratory nitrate reductase (NarGHI; ERIC1_1c24810 -ERIC1_1c24830, ERIC2_c40560 - ERIC2_c40590) are present in the genomes of both strains but complete general nitrite reductase genes could be found only in genome of DSM 25430 (NasDE; ERIC2_c25390 - ERIC2_c25400).

#### Sugar metabolism

At the beginning of the infectious process, during the non-invasive phase *P. larvae* proliferates in the midgut of the larvae [8] and lives on the incoming larval diet. The diet of worker and drone larvae changes over time from pure royal jelly (RJ) to a mixture of RJ, honey, and pollen while queen larvae are fed RJ throughout their entire larval development [25]. In any case, crude proteins (12.5% in RJ) [26] and simple sugars (11% in RJ) [26] are the main constituents of the larval diet. Fructose and glucose, which are the dominant sugars of the added honey, can be metabolized by vegetative *P. larvae* [12]. Our genome analysis revealed that *P. larvae* metabolizes D-glucose and D-fructose mainly via the Embden-Meyerhof-Parnas (EMP) and oxidative pentose-phosphate pathway, as a complete set of genes for the conversion of glucose 6-phosphate to pyruvate was present and genes coding for a 1-phoshofructokinase (ERIC1_1c31490; ERIC2_c36850) and 6-phosphofructokinase (ERIC1_1c16820; ERIC2_c11750) were found. The genomes of both sequenced strains also revealed a possible mechanism for the uptake and catabolism of trehalose, the main carbohydrate in honey bee larval hemolymph [27]. Both strains are equipped with a putative trehalose specific II^c^ component of a PTS system (ERIC1_1c04630; ERIC2_c17680) for uptake of the disaccharide. A regulated uptake system for trehalose might correspond to the second, the invasive phase of infection. In this phase when the bacteria invade the haemocoel [8] and start to consume and degrade the entire larval biomass, disaccharides like trehalose become available.

### Toxins

Bacterial pathogens need a diverse repertoire of genes providing them with unique mechanisms to colonize the host and escape the host’s immune system. These genes and the corresponding gene products conferring the pathogenic phenotype can be summarized as virulence genes and virulence factors, respectively. Potential virulence-associated determinants of both *P. larvae* genotypes ERIC I and ERIC II were identified *in silico* based on sequence similarity to known microbial virulence factors.

#### AB Toxin loci

**Figure 2:**
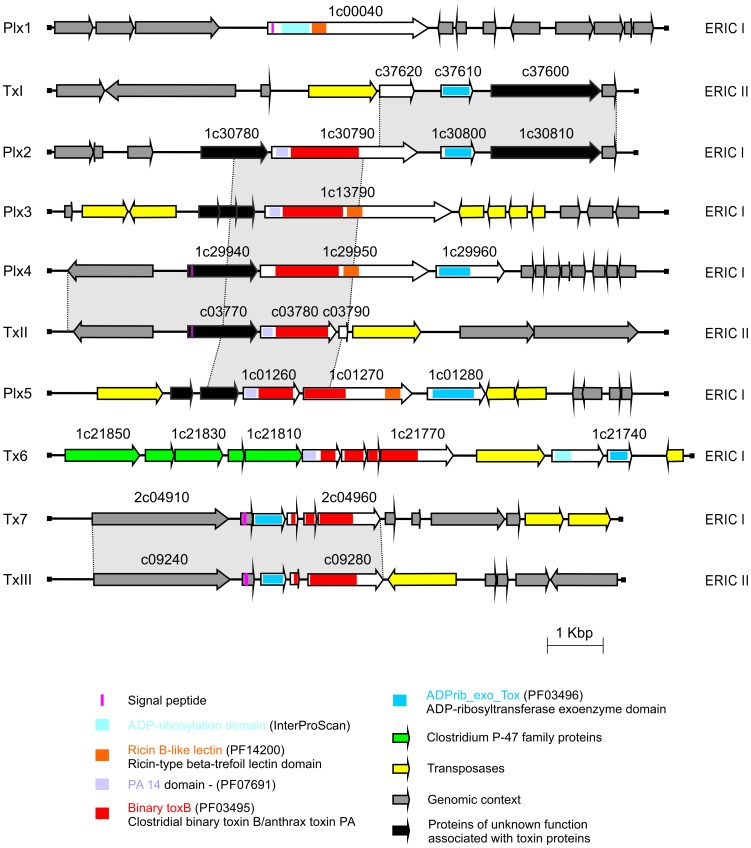
Genetic organization of toxin complex loci identified in *P. larvae* DSM 25719 and DSM 25430. Related ORFs are shown in the same colors. Toxin loci Plx1-7 are encoded within *P. larvae* DSM 25719 genome (ERIC I) and toxin loci TxI-III within *P. larvae* DSM 25430 (ERIC II).

It was recently demonstrated that breaching of the larval midgut epithelium and invasion of the haemocoel is a crucial step in *P. larvae* pathogenesis. It was suggested that the observed changes in epithelial cell morphology during this process are the result of toxin activity [8]. In accordance, putative AB toxin gene fragments have been identified in ERIC I strains by subtractive suppression hybridization [28]. This result could recently be supported during the draft annotation of the 646 contigs of the fragmented *P. larvae* genome sequence. The existence of allegedly sixteen toxin proteins was suggested [20], [21]. Here, we present the definite identification of seven toxin encoding loci (Plx1-7) in the genome of ERIC I genotype strain DSM 25719 (Figure 2) with five of them coding for putatively functional toxins (Plx1-5) (Figure 2). All five putatively functional gene products show similarity to the family of AB toxins known from several other Gram positive and spore-forming bacteria such as pathogenic clostridia and bacilli. AB toxins consist of two subunits (A and B) and display a synergistic binary mechanism for attacking eukaryotic cells. The A subunit possesses enzyme activity and inhibits normal cell functions. The B subunit mediates membrane-binding and transport of the A domain into the host cell (for a recent review: [29]). Based on comparative genome analysis via suppression subtractive hybridization [28] and the application of recently developed molecular tools for *P. larvae* [30], two of the five putatively functional *P. larvae* toxins, Plx1 and Plx2, have already been characterized in detail [31]. Plx1 (Accession No. KC456421; ERIC1_1c00040) is a single-chain AB toxin and belongs to an enigmatic family of toxins [31], so far comprising only few members, the larvicidal toxin MTX1 expressed by *Lysinibacillus sphaericus* and several pierisin-like toxins expressed by *Pieridae*, a large family of butterflies [32], [33]. It has been proposed that Plx1 has ADP ribosyltransferase activity [31] as already shown for MTX1 and pierisin-1 [32], [33]. Plx2 was shown to be a binary AB toxin with two separate ORFs encoding the A and B subunits [31]. The A subunit (Plx2A, ERIC1_1c30800) showed similarity to C3-like Rho-ADP-ribosylating toxins whereas the B subunit (Plx2B, ERIC1_1c30790) showed similarity to the B subunit of the C2 binary toxin of *Clostridium botulinum* [31]. Exposure bioassays performed with wildtype *P. larvae* and corresponding knock-out mutants lacking Plx1 or Plx2 expression revealed that both toxins are important virulence factors for *P. larvae* ERIC I [31].

Two other toxin loci in the genome of *P. larvae* ERIC I (Plx4, and Plx5) also encode binary AB toxins with separate ORFs coding for “A” domains and “B” domains. The “B” domains (ERIC1_1c29950, ERIC1_1c01270) are located upstream of the “A”-domains (ERIC1_1c29960, ERIC1_1c01280). The two predicted “A” domains show similarity to ADP ribosyltransferases of *B. cereus* or *B. thuringiensis* whereas the predicted “B” domains show similarity to the “B” domains of clostridial enterotoxic C2 toxin of *Clostridium botulinum* or toxin CDT of *C. difficile* (for a recent review on binary toxins see [29]). The combination of *Clostridium*-like translocation domains with *B. cereus*-like ADP ribosyltranferases is unique and indicates that *P. larvae* developed its own specific mechanisms to intoxicate honey bee larval cells.

**Figure 3:**
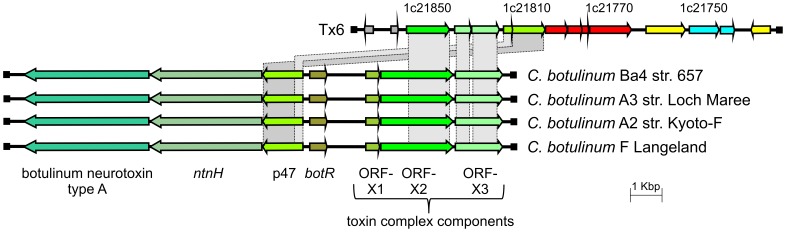
Comparison of the *P. larvae* DSM 25719 Tx6 toxin-encoding locus with selected strains. Related ORFs are shown in the same following colors: yellow, transposases; grey, genomic context; green, botulinum toxin complex components; cyan, binary toxin A domain-containing protein; and red, binary toxin B domain-containing proteins. The arrows indicate the direction of transcription.

Two *P. larvae* ERIC I toxin loci (Tx6, Tx7) harbor only remnants of toxin genes. In Tx6, a putative B domain gene is interrupted by three mutations splitting the gene into several ORFs (ERIC1_1c21800 to ERIC1_1c21770); the upstream located putative A domain gene is interrupted by one mutation resulting in two ORFs (ERIC1_1c21750 - ERIC1_1c21740). In addition, a transposase is inserted between the genes encoding B and A domain. These mutations indicate that the AB toxin gene cluster is non-functional. Downstream of this cluster, remnants of a *Clostridium botulinum* neurotoxin type A gene cluster [34] are located (Figure 3). ORF X2, ORF X3 (interrupted by a stop codon), and p47 are present whereas ORF X1, the botulinum neurotoxin and *ntnH* genes are missing (Figure 3). The existence of these genes suggests horizontal gene transfer from the food-borne pathogen *C. botulinum* to *P. larvae*. Correspondingly, *C. botulinum* can frequently be found in the beehive environment and especially in wax and honey [35], [36]. One explanation for the loss of function in *P. larvae* is that the neurotoxin type A gene cluster coding for a vertebrate toxin may not confer any fitness increase for entomopathogenic *P. larvae*. In Tx7, a B domain gene, which is interrupted by two mutations resulting in three ORFs, is located upstream of a putative gene encoding an A domain. Although the putative A domain gene appears functional, it is unlikely that this gene cluster codes for a functional AB toxin. However, the formation of functional toxin complexes involving the Tx7 A domain might still be possible by interaction of the A domain with a B domain encoded by another toxin locus.

In the genome of *P. larvae* DSM 25430 (genotype ERIC II), we identified five toxin-encoding loci and none of them harbored a putatively functional toxin gene. Sequence comparison revealed that TxI, TxII, and TxIII of DSM 25430 are remnants of Plx2, Plx4, and Tx7 loci of DSM 25719 (Figure 2). Additionally, DSM 25430 encodes small fragments of toxins (ERIC2_c05300, ERIC2_c05310, ERIC2_c33880). Several mutations including insertion of transposases and deletions of gene fragments in DSM 25430 resulted in toxin loci, which only harbor footprints of formerly functional toxin gene clusters. Thus, although highly virulent on individual larval level [7], strain DSM 25430 lacked classical toxins raising the interesting question of how this bacterium is killing honey bee larvae.

Many entomopathogenic bacteria like *B. thuringiensis*, *Brevibacillus laterosporus* and *Lysinibacillus sphaericus* produce large crystalline inclusions that consist of entomocidal protein protoxins [37]. These parasporal crystal proteins (also named cry-toxins or δ-endotoxins) are activated upon ingestion through proteolytic processing in the midgut environment. Subsequently, pore structures capable of inserting into the host cell membrane and eventually leading to cell death are formed. Although *P. larvae* is an acrystalliferous bacterium, which does not produce δ-endotoxins, four toxin loci in strain DSM 25719 and DSM 25430 contain short ORFs with weak similarity to δ-endotoxins of *B. thuringiensis*. These ORFs are located downstream of the genes coding for the putative “AB” toxins Plx2, Plx3, Plx4, and Plx5 and the B domain gene in TxIII. The role of these ORFs is unclear, as they are too short to code for functional cry-like toxins and some of them are interrupted by insertions/deletions and/or frameshift mutations.

We also identified four clostridial epsilon toxin ETX/*Bacillus* mosquitocidal toxin MTX2 gene homologs in the genome of DSM 25719 (ERIC1_1c06220, ERIC1_1c16820, ERIC1_1c13570, ERIC1_1c09720). Comparison of the predicted amino acid sequences revealed 31% identity to epsilon toxin type B of *Clostridium perfringens* (Q02307) and 29% identity to a novel mosquitocidal toxin Mtx2 encoded by *L. sphaericus* (Accession Q45422). Mtx2 is unrelated to AB toxin MTX1 but shares regions of similarity with epsilon toxin type B of *C. perfringens*. Epsilon toxin is the major virulence factor of *C. perfringens* types B and D. This microorganism is responsible for fatal enterotoxaemia in animals, mainly in lambs and goat, and more rarely in cattle [38]. The functionality and role of these putative epsilon toxin homologs in *P. larvae* DSM 25719 need to be established.

In *P. larvae* DSM 25430, one ORF (ERIC2_c11360) corresponding to the Mtx2 homolog (ERIC1_1c06220) of DSM 25719 could be identified. However, insertion of a transposase at the 5′-end destroyed the 5′-region of the gene including the start codon. Therefore, it is unlikely that a functional epsilon toxin homolog exists in strain DSM 25430.

In conclusion, we identified several putative functional toxin-encoding genes and gene clusters in the genome of DSM 25719, but none in the genome of DSM 25430. The genome of DSM 25430 harbored several toxin loci that show similarity to corresponding loci in the genome of DSM 25719, but these were non-functional. These results confirmed previous findings obtained by subtractive suppression hybridization (SSH) that failed to identify any toxin gene in *P. larvae* genotype ERIC II but already suggested the existence of AB toxins in ERIC I [28]. These results are surprising, since ERIC II is more virulent than ERIC I on the larval level and kills larvae faster than ERIC I [5]. This tremendous difference between the two genotypes indicates that they developed completely different modes of pathogenesis or the genes, which encode the lethal factors for the honey bee have not been identified yet. While ERIC I might still rely on toxins for killing larvae, ERIC II became independent from toxins most likely by acquiring other virulence factors such as the recently identified ERIC II-specific S layer protein SplA [30].

#### Cytolysins, iron acquisition

The genomes of *P. larvae* DSM 25719 and DSM 25430 harbored four (ERIC1_1c07340, ERIC1_2c04780, ERIC1_1c35160, ERIC1_2c04240) and two (ERIC2_c15500, ERIC2_c32790) genes, which were predicted by similarity-based annotation as hemolysin domains. Hemolysins, or more correctly cytolysins, are a group of membrane-damaging toxins that disrupt host cell membranes either enzymatically (by the means of proteases or phospholipases) or by forming pores in the host cell membrane (pore-forming toxins, PFTs). However, the highest similarity of the proteins ERIC1_1c07340, ERIC1_2c04780, ERIC1_1c35160 and ERIC2_c15500 point to the hemolysin III-related protein family whereas ERIC1_2c04240 and ERIC2_c32790 cluster within the pore-forming cytolysin TlyA protein family. Apparently these cytolysins represent members of a new *Paenibacillus*-specific protein family and may play an important role in bacterial pathogenesis. It has been reported that the related cytolysins directly act on certain mammalian cells as cytotoxic virulence factors or are responsible for iron acquisition during bacterial growth. It has been shown that hemolysin III of *B. cereus* is a pore-forming, haemolytic cytolysin [39]. Iron acquisition during growth in mammalian as well as insect hosts poses specific difficulties for bacterial pathogens and thus might be the reason for the acquisition of related toxin genes.

The availability of iron within extracellular fluid is highly restricted. Bacteria living inside their hosts often need to establish methods for extracting the metal from host proteins. Iron chelators (siderophores) are produced and secreted specifically in response to iron deficiency. The genome of *P. larvae* DSM 25719 encodes two iron ABC transport systems (ERIC1_1c03900-ERIC1_1c03960 and ERIC1_1c31350-ERIC1_1c31370) whereas DSM 25430 encodes only one (ERIC2_c36980-ERIC2_c37000). The gene ERIC1_1c03900 showed similarity to the iron-regulated surface determinant (*isd*) system, which is used by *Staphylococci* to bind hemoproteins, remove the heme molecule, and transport heme into the bacterial cytoplasm. Its function in *P. larvae* during larval infection needs to be established. Additionally, both strains contain genes for a ferrous iron transport cluster, which encode a FeoA family protein (ERIC1_1c32860 and ERIC2_c35360) and the ferrous iron uptake protein B (ERIC1_1c32870, ERIC2_c35350).

### Proteases, Collagenases, Chitinases

Proteases have been discussed as key virulence factors of *P. larvae* since decades [16], [40], [41]. We found 159 full or truncated proteases in the genome of DSM 25719 and 128 in that of DSM 25430, which belong to different families (Table S1, Table S2) [42]. Some of these enzymes might be involved in disruption of the epithelial barrier integrity of honey bee larvae by degrading cell-cell and cell-matrix junctional structures.

The main structural component of the extracellular matrix is collagen. To destroy this barrier bacteria secrete enzymes degrading the major matrix components such as collagenases, hyaluronidases and proteases [43]. Collagenases have been widely used for the disintegration of connective tissue and separation of tissue culture cells, because of the broad substrate specificity [44]. We identified putative genes encoding collagenases, which belong to two different families (Table S1, Table S2). Microbial collagenases family 9 have been identified from bacteria belonging to the genera *Vibrio* and *Clostridium* [45], [46]. Collagenase is used to degrade the collagen barrier of the host during invasion. Peptidases family M9 (ERIC1_1c24570, ERIC2_c40370), and peptidases belonging to family U32 (ERIC1_1c37420, ERIC1_1c37430, ERIC2_c05010, ERIC2_c27620, and ERIC2_c27630) were identified in both genomes. Glycosaminoglycans, like hyaluronan and chondroitin, are polymers built of hexosamine uronic acid disaccharide units and are a major component of the extracellular matrix [47]. We identified one putative polysaccharide lyase family 8 protein in each genome (ERIC1_1c09810, ERIC2_c23740). Polysaccharide lyase family 8 consists of a group of secreted bacterial lyase enzymes, e.g. hyaluronidases, which are able to degrade hyaluronan, chondroitin, and chondroitin sulfates [48]. Hyaluronidases are also known as virulence factors [49], as they are able to degrade the connective tissue of eukaryotes [50].

#### Chitinases

The peritrophic matrix (PM) represents the first barrier for the bacteria to reach the epithelium [9]. The PM is a chitin and glycoprotein layer that borders the larval midgut and protects the midgut epithelium from abrasive food particles, digestive enzymes and pathogen infections [51]. In both analyzed genomes, we found genes with chitin-binding domains (ERIC1_3c00760, ERIC1_1c15380; ERIC2_c22220 and ERIC2_c15060), which might aid in chitin degradation. Additionally, a region splitted in several ORFs and containing a chitinase A (GH18 family) N-terminal domain (ERIC2_c09520) was identified in both genomes. The protein sequences deduced from these pseudogenes showed significant protein sequence similarity with putative *C. botulinum* chitodextrinase [52]. However, although all *Paenibacillus* species genomes sequenced so far contain several chitinase genes, no entire and putatively functional chitinase gene could be detected in the genomes of *P. larvae* DSM 25719 and DSM 25430 posing the intriguing question how the described chitin-degradation by *P. larvae* during infection [9] is achieved.

#### Enhancin-like protease

In the genome of DSM 25719 and DSM 25430 genes encoding a metalloendopeptidase of the enhancin family were present. Enhancin was originally described for granuloviruses (GVs) and plays an important role in viral infection. Enhancin is incorporated into viral occlusion bodies, which are ingested by the host. The occlusion bodies are broken down in the midgut of the host and the enhancin is released. Subsequently, enhancin disrupts the protective peritrophic matrix (PM), allowing the virion to enter the epithelial cells of the insect gut. The PM has a lattice structure formed by chitin and insect intestinal mucin (IIM), and the viral enhancin protein targets the IIM for degradation [53], [54]. A similar mode of action has been described for the bacterial enhancin-like protein of *Bacillus thuringiensis* (Bel) that exhibits 20 to 30% amino acid identity to viral enhancin proteins and 95% identity to enhancin-like proteins from other bacteria such as *Yersinia pestis*, *B. anthracis*, and *B. cereus* [55]. Thus, enhancin-like proteases from *P. larvae* might enhance bacterial infection by degradation of the peritrophic matrix (PM) of the insect midgut. However, the orthologous genes in *P. larvae* DSM 25719 (ERIC1_1c37500/ERIC1_1c37520) and *P. larvae* DSM 25430 (ERIC2_c09380-ERIC2_c09400) are dysfunctional due to insertion of transposases or frameshift mutations.

#### Serine proteases

**Figure 4:**
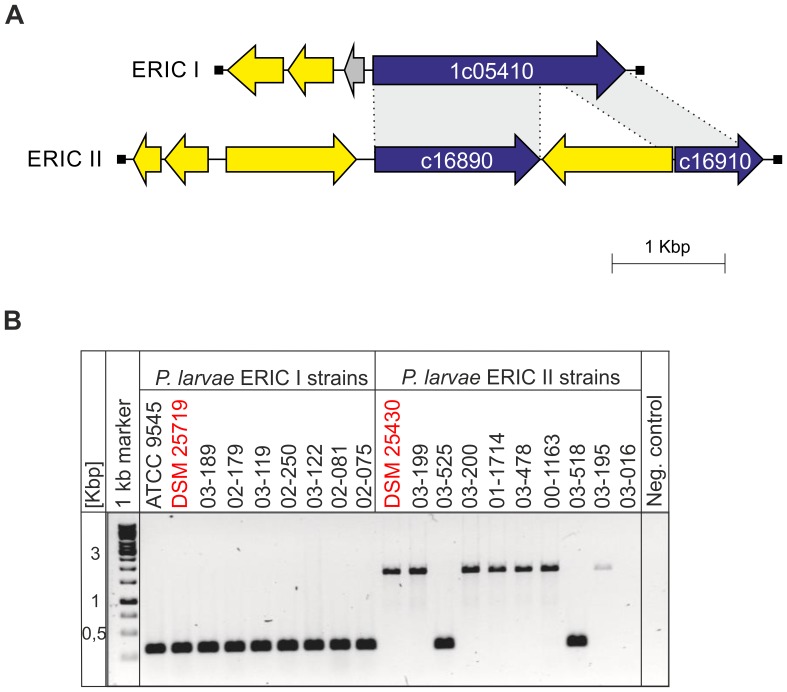
Genetic organization of subtilisin-like serine protease gene locus. Related ORFs are shown in the same following colors: blue, subtilisin-like serine protease gene; yellow, transposases/integrases; grey, genomic context (A). The similarities between pairs of sequences are depicted. PCR screening of ERIC I and ERIC II strains for functional or disrupted subtilisin-like serine protease genes (B).

Serine proteases are ubiquitous enzymes with a nucleophilic Ser residue at the active site and believed to constitute nearly one-third of all the known proteolytic enzymes. They function in diverse biological processes such as digestion, blood clotting, fertilization, development, complement activation, pathogenesis, apoptosis, immune response, secondary metabolism, with imbalances causing diseases like arthritis and tumors [56], [57]. The genomes of *P. larvae* DSM 25719 and *P. larvae* DSM 25430 contain 11 and 6 genes coding for family S8 peptidases, respectively (Table S1, Table S2). Beside the additional proteases of DSM 25719 some of the remaining have no ortholog within the DSM 25430 genome. For instance *P. larvae* DSM 25719 gene ERIC1_1c21520 no ortholog could be found in the sequenced *P. larvae* DSM 25430 genome and ortholog ERIC1_1c05410 gene is interrupted into two pseudogenes (ERIC2_c16890-ERIC2_c16910) by a transposase in the sequenced DSM 25430 genome (Figure 4A). All tested *P. larvae* ERIC I-strains harbored these two serine protease genes. The serine protease of DSM 25430 is probably non-functional due to an inserted transposase. To test whether or not all strains of *P. larvae* ERIC II indeed lack a functional serine protease, we screened a collection of ERIC II strains for the inserted transposase: one out of ten tested strains did not give any signal for this gene while seven of the tested strains carried the inserted transposase and another two still harbored the non-disrupted serine protease gene (Figure 4B). Therefore, the observed inactivation of this serine protease gene is strain-specific within the *P. larvae* genotype ERIC II.

#### Proteases for escaping immune response

Infected larvae activate an immune response already early during infection by up-regulating expression of toll receptors, antimicrobial peptides and lysozyme [55], [58], [59]. However, rescue of infected larvae by immune response has never been observed, suggesting that *P. larvae* can counteract the larval immune defense quite efficiently. We identified virulence-associated genes, which might be involved in this function. In *P. larvae* DSM 25430, the gene ERIC2_c27330 encodes a metalloprotease that belongs to the M6 peptidase family. Interestingly, this protein shows significant amino acid identity (41%) to the immune inhibitor A precursor (InhA), a virulence factor encoded by the *inhA* gene of *B. thuringiensis*. InhA of *B. thuringiensis* specifically cleaves antibacterial peptides produced by insect hosts [60]. We hypothesize that the putative *P. larvae* InhA has a similar function and helps *P. larvae* to survive the larval immune response. However, this gene (ERIC1_1c15040) is interrupted by a frameshift mutation in all *P. larvae* ERIC I strains analyzed so far, suggesting that it is non-functional in ERIC I genotype.

### Secondary Metabolites

**Figure 5:**
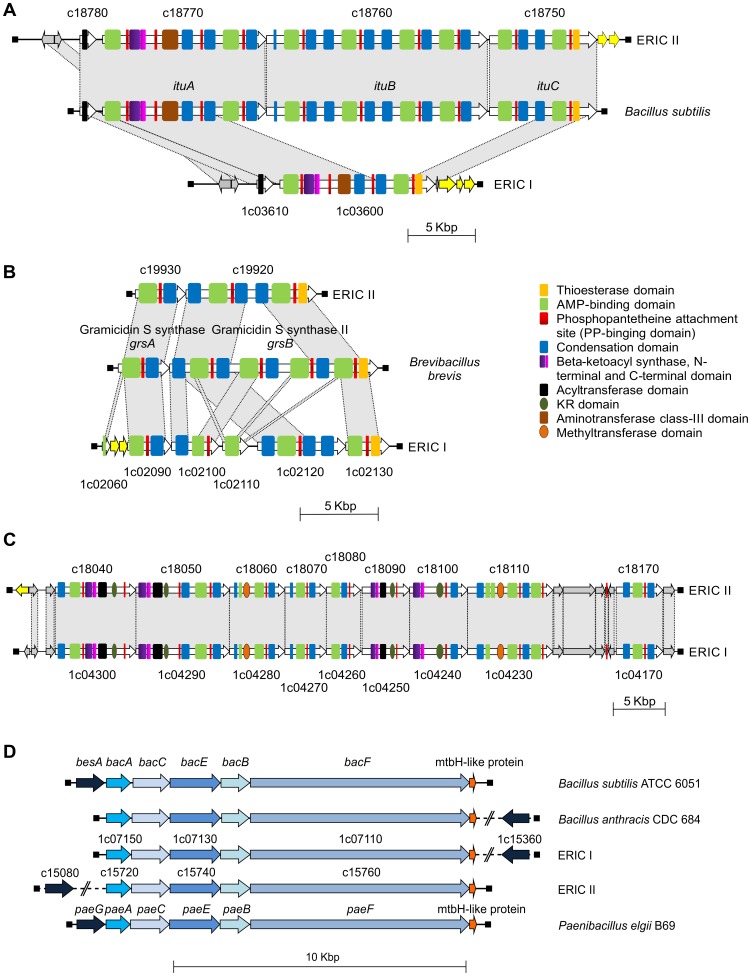
Genetic organization of *P. larvae* PKS/NRPS biosynthesis gene clusters. Shown are the PKS/NRPS biosynthesis gene clusters from *P. larvae* DSM 25719 (ERIC I) and DSM 25430 (ERIC II) genes. Iturin family lipopeptide antibiotic NRPS/PKS cluster (A), gramicidin S synthase NRPS cluster (B), organization and architecture of a novel NRPS/PKS cluster in ERIC I and ERIC II (C), and the bacillibactin/paenibactin NRPS cluster (D). Related ORFs are shown in the same colors.

Microorganisms are often capable of producing metabolites, which have a secondary role in self-defense or aggression [61]. Polyketide synthases (PKS) and nonribosomal peptide synthetases (NRPS) are the producers of two large groups of natural products with remarkable structural diversity and biological activities, including antibiotic, antifungal, anticancer, immunosuppressant and cholesterol-lowering activities [62]. We recently presented evidence for the existence of NRPS-PKS clusters in the genomes of all *P. larvae* genotypes [28]. In the sequenced genomes, we identified four different NRPS or PKS clusters (Figure 5). Both genomes seem to encode two PKS-NRPS hybrid clusters and two NRPS clusters. Three of these clusters could be assigned with a putative function; the biological role of the other cluster remains unknown.

The first NRPS/PKS cluster with a predicted function (strain DSM 25430, Figure 5A) is putatively encoding an iturin family lipopeptide antibiotic. Iturin family antibiotics are heptapeptides with a β-amino fatty acid that show strong antifungal activity [63], [64]. For example, iturin A destroys the fungal cytoplasmic membrane, which leads to transmembrane channels, permitting the release of vital ions such as K^+^ from the fungal cells. While the antibacterial activities of the iturin family antibiotics are limited [65], a lytic activity against human erythrocytes could be detected [66]. ORF arrangement and the domain organizations showed a high similarity to mycosubtilin, iturin A and bacillomycin synthetase [67], [68]. The putative DSM 25430 operon spanned 37 kb. We suggest that this putative iturin family synthetase mainly exhibits antifungal and antibacterial activity, but lytic activity against hemocytes in the hemolymph of the honey bee larvae is also conceivable [69]. However, the corresponding DSM 25719 region was significantly shorter (12.5 kb).

The second NRPS cluster found in both genomes showed similarity to the gramicidin S synthetase (Figure 5B). Gramicidin S is a potent cyclopeptide antibiotic, as it interacts with the cell membrane of target microorganisms and disrupts it [70]. The third large NRPS/PKS-cluster was also present in both strains (Figure 5C). It comprised approximately 60 kb and exhibited no similarity to known NRPS/PKS clusters.

Expression of the fourth NRPS cluster will putatively result in the production of a siderophore with similarity to bacillibactin. Siderophores are low-molecular mass microbial compounds with a very high affinity for iron [71], especially Fe^3+^ [72]. The genes involved in the biosynthetic pathway for bacillibactin in *B. subtilis* have been characterized [73] and compared with paenibactin, a catecholate siderophore produced by *Paenibacillus elgii* B69 [74]. The paenibactin gene cluster consisted of six genes (*paeGACEBF*), of which three contained NRPS-domains (*paeE*, *paeB* and *paeF*). The genomes of DSM 25719 encoded gene clusters, which showed high similarity to the paenibactin gene cluster of *P. elgii* B69 (Figure 5D).

**Figure 6:**
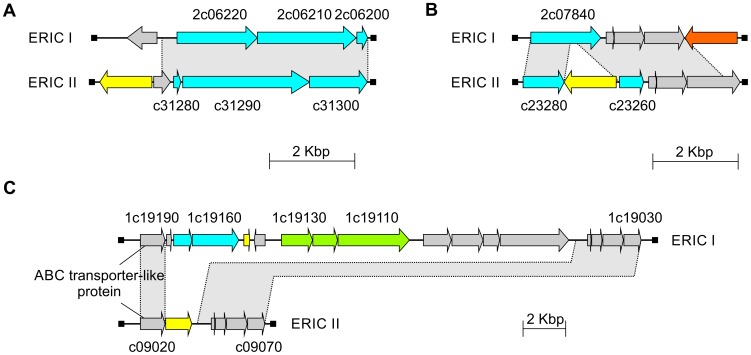
Genetic organization of lantibiotic biosynthesis clusters from *P. larvae* DSM 25719 (ERIC I) and DSM 25430 (ERIC II). Related ORFs are shown in the same following colors: yellow, transposases; grey, genome context; green, type I restriction system; cyan, lantibiotic biosynthesis clusters; and orange, integrase. The arrows indicate the direction of transcription.

In the genomes of DSM 25719 and DSM 25430, we also identified ORFs coding for proteins with high similarity to a lanthionine synthetase, suggesting that *P. larvae* produces lantibiotics. Lantibiotics are a unique class of peptide antibiotics. Lantibiotics are small antimicrobial agents (19–38 amino acids) derived from ribosomally synthesized peptides. They are produced by Firmicutes, and include mutacin, subtilin, and nisin. Many lantibiotics are bacteriocidal against a variety of Gram positive bacteria at nanomolar levels [75]. In the genome of DSM 25719, we found three lantibiotic biosynthesis clusters (Figure 6). Due to this genomic difference between DSM 25719 and DSM 25430, suppression subtractive hybridization analysis was successful in already predicting the potential for the production of lantibiotics at least for DSM 25719 [28].

### Clustered Regulatory Interspaced Short Palindromic Repeats (CRISPRs)

CRISPRs systems are genomic systems for host defense against invasive nucleotide sequences such as phages and plasmids. CRISPRs consist of a genomic repeat region, the CRISPR in *sensu stricto* and associated proteins the CRISPR-associated proteins (CAS; CRISPR/Cas) or Cas module-RAMP (Repeat-Associated Mysterious Proteins) systems (CRISPR/Cmr). A CRISPR locus is a class of direct repeats, which contain unique, target specific spacer sequences of similar length located between each pair of repeats [76], [77]. Genome analysis showed the presence of seven possible CRISPRs candidates in ERIC I whereas in ERIC II only one candidate was found [78] (Table S4, Table S5).

In the genome of DSM 25719 CRISPR/Cas cluster was found (ERIC1_2c05680-ERIC1_2c05750) and comparative analysis showed that it was DSM 25719-specific. Downstream of ERIC1_2c05750 gene we identified five direct repeats and the transposase. Additionally, a CRISPR/Cmr cluster was found in both sequenced strains (DSM 25719: ERIC1_1c30330-ERIC1_1c30350; and DSM 25430: ERIC2_c04120-ERIC2_c04180). However, in strain DSM 25719 genes *cmr5* and *cmr6* are missing. In both strains this cluster is flanked by transposases. It has been suggested that CRISPRs play a role in chromosomal rearrangement [79] and appear to be among the most rapidly evolving elements in the genome. Closely related species and strains differ in their CRISPR composition [80], [81].

### Pathogenicity Islands (PAIs)

To identify orthologous genes, as well as strain/genotype-specific gene content a bidirectional BLAST was employed. DSM 25719 and DSM 25430 genomes share a large core genome. Island-like genomic regions (GI) encoding a variety of putative virulence-associated and fitness-associated traits were identified. The DSM 25719 genome contained 23 regions larger than 5 kb, excluding predicted prophage regions (Figure 2, Table S6). Most of the genes within the DSM 25719-specific regions encoded proteins of unknown function or hypothetical proteins. Nevertheless, a significant number of mobile elements such as insertion elements, transposases, integrases, and recombinases were identified within each region. The above-described toxin loci in general belong to the *P. larvae* ERIC I-specific gene pool.

The largest DSM 25719-specific region (GI21) spanned over 120 kb and comprised the genes ERIC1_2c04540 (integrase family protein) to ERIC1_2c05970. This region encoded a *cas* operon, subtilisin E, ferrous iron transport proteins, amino acid permease, putative transporter proteins, and toxin locus Tx7. GI5 (ORFs: ERIC1_1c03660-ERIC1_1c04000) encoded 35 predicted proteins, including putative O-methyltransferase, type-2 restriction enzyme *Bsu*BI and iron transport system *isd*. GI19 represents the DNA region (21 kb) ranging from ERIC1_2c02250 to ERIC1_2c02370. It harbored 13 predicted proteins with similarities to RHS repeat-associated core domain-containing protein, secreted proteins, and cell surface proteins. GI10 (ERIC1_1c19060- ERIC1_1c19180) encoded 13 ORFs, including SMC domain protein, type I restriction-modification system and a lantibiotic-modifying enzyme. These ORFs were absent in the DSM 25430 genome. Above described toxin loci could also be identified within DSM 25719-specific genomic regions such as toxin locus Plx1 within GI1, and toxin loci Plx5, Plx3, Tx6 within GI2, GI8, and GI13, respectively.

In contrast to DSM 25719 the DSM 25430 genome contained only three *P. larvae* ERIC II-specific regions that exceeded 5 kb, excluding putative phage regions (Table S7). The largest DSM 25430-specific region (GI2) spanned over 25 kb, comprised the genes ERIC2_c18730 to ERIC2_c18760, and harbored genes with similarity to iturin A biosynthesis cluster. Only part of the cluster could be found within the DSM 25719 genome. The two other genomic regions encoded insertion elements and hypothetical proteins but also enzymes like amidinotransferase, alpha/beta hydrolase, monogalactosyldiacylglycerol synthase (GI1) or bacitracin export ATP-binding protein (GI3).

## Conclusions

**Figure 7:**
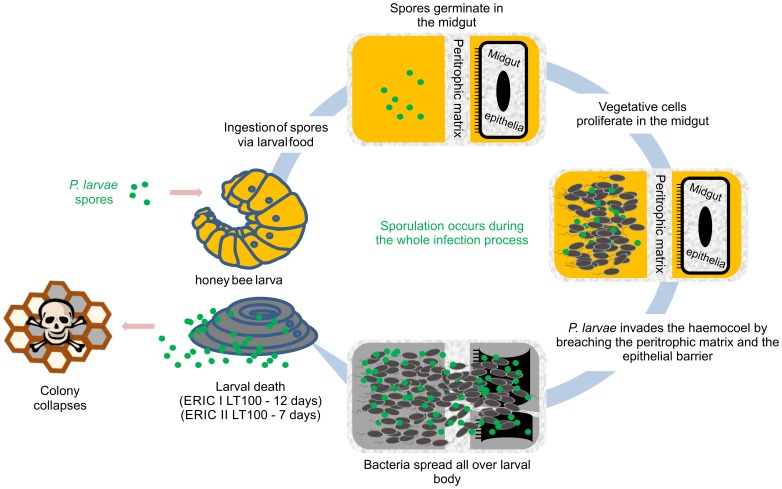
*P. larvae* infection model.

It has been shown that the bacterial life cycle in infected larvae can be divided into two stages (Figure 7) [8]. The genome analysis identified genes, which may encode for all crucial steps of the known life cycle. The early phase of infection is non-invasive and includes ingestion of spore-contaminated food, and subsequently spore germination and proliferation in the midgut lumen. The vegetative bacteria proliferate massively in the midgut lumen prior to breaching the epithelium. During this non-invasive stage *P. larvae* can be considered a commensal bacterium living from the content of the larval diet, i.e., sugars like glucose and fructose. Indeed, *P. larvae* is able to metabolize different sugars and sugar derivatives [12], [82] through several metabolic pathways identified in this study. Although *P. larvae* does not actively attack the infected larvae at this stage of infection, the bacteria are living on the expense of the larvae by competing for incoming food. Therefore, it is not surprising that a recent study of infected larvae using comparative proteomics revealed that infected larvae express higher levels of mitochondrial metabolic enzymes and deplete their energy stores during infection [55]. Thus an enhanced energy demand of infected larvae compared to non-infected larvae is indicated.

The non-invasive phase is followed by penetration of the midgut epithelium and subsequent invasion of the haemocoel via the paracellular route by sequentially destroying the peritrophic matrix, cell-cell junctions, the extracellular matrix, and the larval remains [8]. Bacterial factors likely to be involved in this process are different toxins and secreted extracellular proteases. The extraordinary proteolytic capacity of *P. larvae* has been the topic of many studies [16], [41], [83], [84]. In both genotypes, our DNA sequence-based gene prediction supports the existence of a high number of proteases, which might be involved in several mechanisms during the invasive stage. (i) Proteases able to degrade antimicrobial peptides might neutralize the local immune response mounted by the epithelial cells and help *P. larvae* to survive in the midgut lumen and attack the epithelial barrier. (ii) Once the bacteria have access to the epithelium, proteases, possibly in conjunction with toxins, might be responsible for the disruption of the epithelial barrier integrity by degrading cell-cell and cell-matrix junctions. (iii) Collagenases are then needed to degrade the basement membrane and facilitate access to the underlying tissues. (iv) Once the larvae are dead, proteases are further needed for the subsequent degradation of the larval remains (ropy stage). Pure cultures of *P. larvae* can be obtained from dead larvae at this stage of the pathogenic process [85], a fact that can now be explained by the expression of potent antibiotics as identified in the genomes of both *P. larvae* genotypes. Vegetative bacteria then undergo sporulation and the formed spores drive disease transmission within the colony when disseminated to and ingested by the next larvae.

We have answered many questions with respect to the molecular pathogenesis of *P. larvae* by identifying several novel and important virulence factors of *P. larvae* involved in different steps in pathogenesis. However, our data also raise a new question: It has been shown that *P. larvae* genotype ERIC II is more virulent on the larval level than genotype ERIC I. Surprisingly, we now discovered that the toxins identified in ERIC I (strain DSM 25719) are lacking in the genome of ERIC II (DSM 25430). DSM 25430 strain only harbors non-functional remnants of toxin orthologs found in DSM 25719. In addition, many ERIC II strains lack several proteases (enhancin-like protease, serine proteases), which might also be of central importance for pathogenesis. Considering the high number of predicted genes with unknown function in both genomes (2133 in DSM 25719 and 1400 in DSM 25430) it is possible that unknown toxins or some other novel virulence factor are encoded by these genes. Our genome analysis showed that, although both genotypes are lethal for infected larvae and degrade the larval remains to a ropy mass, they obviously developed different modes of attacking and killing honey bee larvae. Understanding these differences in pathogenesis and elucidating the different virulence mechanisms is a prerequisite for the development of specific treatments against both *P. larvae* genotypes.

## Materials and Methods

### Bacterial Strains and Culture Conditions

The strains used in this study are given in Table S3. *Paenibacillus larvae* strains DSM 25719 and DSM 25430 were streaked out on Columbia sheep blood agar plates and incubated at 37°C for 3 days. For starter cultures, 5 ml 2 × MYPGP broth were inoculated with a single colony and incubated for 10–12 hours at 37°C. For main cultures, bacterial cells from the starter culture were diluted sixtyfold in a total volume of 300 ml 2 × MYPGP, incubated at 37°C overnight with moderate shaking until reaching the exponential phase.

### Isolation of Bacterial DNA and Plasmids

DNA isolation was performed using the MasterPure Gram Positive Purification Kit (Epicentre) following the manufacturer’s instructions. Briefly, bacterial cells were harvested, treated with lysozyme, Gram positive cell lysis solution, proteinase K, protein precipitation reagent, and RNase A. DNA was precipitated by isopropanol, washed with ethanol and suspended in 60 µl elution buffer (10 mM Tris-Cl, pH 8.5). The absence or presence of the subtilisin-like serine protease gene and possible mutations were tested by PCR. Briefly, *P. larvae* genomic DNA isolated from different strains (Table S3) was used as template for PCR amplification (primer pair ERC0390F, GATTCCAATTTGATCAACCA and ERC03906R, TCTGCACTGGAGTTAGTGTA) using a thermal cycler My Cycler™ apparatus (Bio-Rad, Munich, Germany) and a PCR kit (Qiagen, Hilden Germany). PCR amplification was performed with an initial heat activation of the HotStarTaq DNA Polymerase (Qiagen, Hilden, Germany) at 95°C for 5 min, followed by 25 cycles of 30 sec denaturation (94°C), 1 min annealing (56°C) and 1 min elongation (72°C), with a final elongation step of 10 min (72°C). Plasmid preparation was performed using the QIAprep Spin Miniprep kit (Qiagen, Hilden, Germany) as recommended by the manufacturer.

### Genome Sequencing, Assembly and Gap Closure

The extracted DNA was used in a combined sequencing approach using a 454 GS-FLX Titanium XL system with Titanium chemistry (Roche Life Science, Mannheim, Germany) and the Genome Analyzer II system (Illumina, San Diego, CA, USA) as recommended by the manufacturers. Resulting reads were assembled into contigs using MIRA software. For *P. larvae* DSM 25719 (ERIC I), shotgun sequencing resulted in 38.35-fold and 56.49-fold coverage from 454 and Illumina reads, respectively. In case of *P. larvae* DSM 25430 (ERIC II), an average coverage of 64.4-fold was determined (30.07-fold 454 coverage and 35.01-fold Illumina coverage). Editing of shotgun sequences and 454 sequences were done by using GAP4, as part of the Staden software package [86]. To solve problems with misassembled regions caused by repetitive sequences and close remaining sequence gaps, PCR reactions, combinatorial multiplex PCRs reactions, fosmid libraries, plasmid libraries, and primer walking with recombinant plasmids were used. PCR reactions have been carried out with the BioXact Kit (Qiagen, Hilden, Germany) and Phusion High Fidelity DNA Polymerase Kit (Thermo Fisher Scientific, Schwerte, Germany) as described by the manufacturers.

### Bioinformatic Tools

Automatic gene prediction and functional annotation of the protein-coding genes were initially carried out with the IMG/ER (Intergrated Microbial Genomes/Expert Review) system [87]. Subsequently, gene prediction and annotation were manually curated by using the Swiss-Prot, TREMBL and InterPro databases. Complete genome comparisons were done using protein-based bidirectional BLAST. The genome sequences reported in this paper have been deposited in the GenBank database under accession numbers ADFW00000000 (DSM 25430) and CP003355-CP003356 (DSM 25719). Phage regions were predicted by employing PHAST (PHAge Search Tool) [22] and manually corrected. Visualization of plasmid comparisons was done with the ACT program from the Sanger Institute (http://www.sanger.ac.uk/) [88].

### RNA Extraction and Transcript Analysis

Total RNA was isolated either from bacteria grown in MYPGP at 37°C during the log-phase of growth or from experimentally infected larvae [5], [7] at day 4 post infection using Qiagen RNeasy Mini Kit according to the manufacturer’s protocol. An additional DNase step (DNase RNase-free, Qiagen) was included to remove bacterial (and larval) genomic DNA. Analysis of mRNA expression was performed using Omniscript RT (Qiagen) and cDNA was prepared from a total of 500 ng of RNA using random hexamer primers. The derived cDNA was used as template in PCR reactions with specific primers.

## Supporting Information

Figure S1 **Analysis of** ***P. larvae*** **plasmids pPLA1_10 and pPLA2_10.**
(PDF)

Table S1 **Peptidases identified and classified in the genome of** ***P. larvae*** **strain DSM 25719.**
(PDF)

Table S2 **Peptidases identified and classified in the genome of** ***P. larvae*** **strain DSM 25430.**
(PDF)

Table S3 ***P. larvae*** **strains used in this study.**
(PDF)

Table S4 **CRISPR analysis of the** ***P. larvae*** **strain DSM 25719 genome.**
(PDF)

Table S5 **CRISPR analysis of the** ***P. larvae*** **strain DSM 25430 genome.**
(PDF)

Table S6 **Strain-specific regions identified in the genome of** ***P. larvae*** **strain DSM 25719.**
(PDF)

Table S7 **Strain-specific regions identified in the genome of** ***P. larvae*** **strain DSM 25430.**
(PDF)
