# Supplementary material for: How to Kill the Honey Bee Larva: Genomic Potential and Virulence Mechanisms of Paenibacillus larvae
Source: PLoS One. 2014 Mar 5;9(3):e90914. doi: 10.1371/journal.pone.0090914 (PMC3944939; doi:10.1371/journal.pone.0090914)
Supplement: Figure S1 — Analysis of P. larvae plasmids pPLA1_10 and pPLA2_10. (PDF) [file pone.0090914.s001.pdf]

A

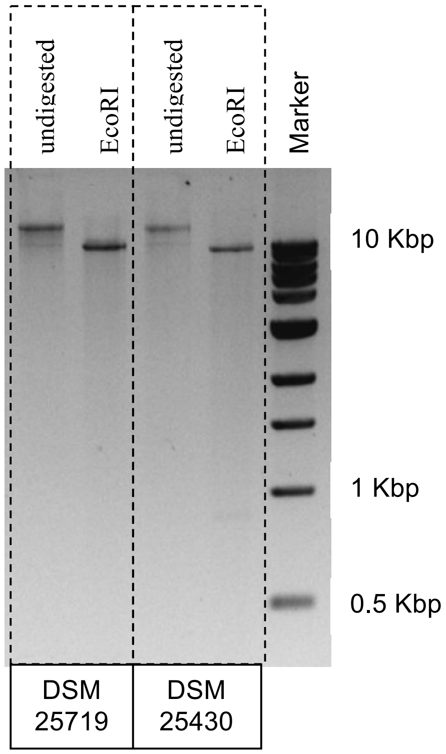

B

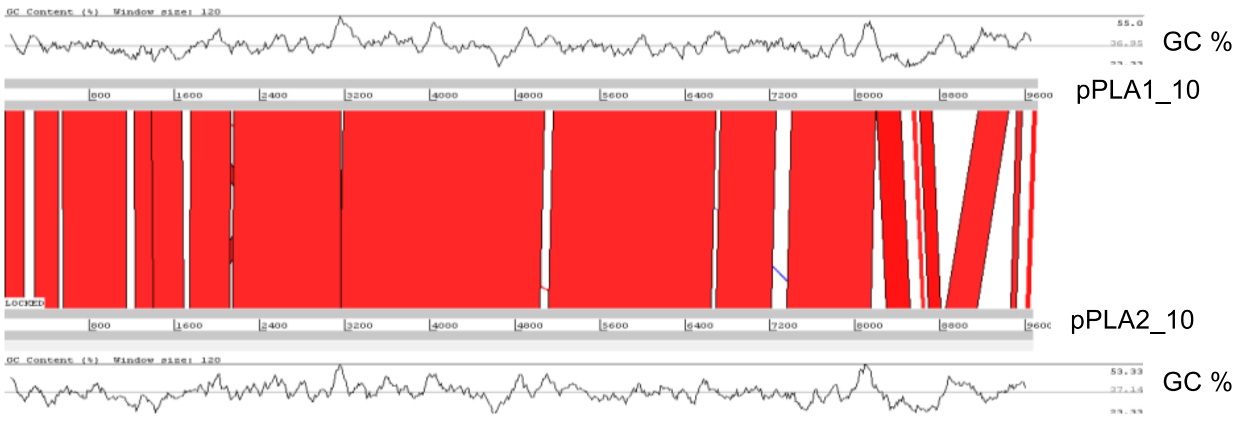

Figure S1. Analysis of *P. larvae* plasmids pPLA1_10 and pPLA2_10.

Restriction analysis of *P. larvae* plasmids pPLA1_10 and pPLA2_10 derived from *P. larvae* strains DSM 25719 and DSM 25430, respectively, (A) and pPLA1_10 and pPLA2_10 sequence comparisons (B). Comparative analysis was performed by employing the ACT software tool [1]. The relationships between each pair of sequences are depicted. Similar coding sequences were indicated by red lines.

Reference

1. Carver TJ, Rutherford KM, Berriman M, Rajandream MA, Barrell BG, Parkhill J (2005) ACT: the artemis comparison tool. Bioinformatics 21: 3422–3423.
