# Supplementary material for: How to Kill the Honey Bee Larva: Genomic Potential and Virulence Mechanisms of Paenibacillus larvae
Source: PLoS One. 2014 Mar 5;9(3):e90914. doi: 10.1371/journal.pone.0090914 (PMC3944939; doi:10.1371/journal.pone.0090914)
Supplement: Table S1 — Peptidases identified and classified in the genome of P. larvae strain DSM 25719. (PDF) [file pone.0090914.s002.pdf]

Table S1. Peptidases identified and classified in the genome of *P. larvae* strain DSM 25719.

Gene ID	MEROPS family [1]	MEROPS entry	E-value*
ERIC1_1c10610	A08	MER070683	2.00E-12
ERIC1_1c39320	A25	MER166302	7.70E-86
ERIC1_1c10550	C26	MER146598	3.10E-89
ERIC1_1c19730	C26	MER061258	1.40E-61
ERIC1_1c23710	C26	MER134379	3.90E-66
ERIC1_1c28490	C26	MER066434	1.90E-06
ERIC1_1c18380	C40	MER003805	2.90E-28
ERIC1_1c18810	C40	MER002450	4.50E-33
ERIC1_1c20510	C40	MER003807	6.00E-18
ERIC1_1c22900	C40	MER003805	1.90E-27
ERIC1_1c25590	C40	MER003807	1.20E-20
ERIC1_1c04050	C44	MER033254	1.40E-20
ERIC1_1c13170	C44	MER033254	8.50E-20
ERIC1_1c19450	C44	MER004101	7.50E-65
ERIC1_1c19840	C44	MER020221	1.30E-28
ERIC1_1c22530	C44	MER003327	1.60E-60
ERIC1_1c36310	C44	MER033254	1.20E-16
ERIC1_1c25870	C56	MER031432	2.70E-11
ERIC1_4c00600	C56	MER002455	3.90E-32
ERIC1_1c03910	C60B	MER020433	1.30E-49
ERIC1_1c12420	C82	MER076166	3.00E-08
ERIC1_2c06260	C82	MER076166	5.70E-05
ERIC1_2c05350	S08A	MER055152	1.90E-65
ERIC1_1c28020	M01	MER055808	1.70E-29
ERIC1_1c16550	M03B	MER001163	6.70E-68
ERIC1_2c07220	M03B	MER084913	8.50E-64
ERIC1_1c04550	M04	MER001026	6.70E-104
ERIC1_1c13440	M04	MER001026	3.90E-87
ERIC1_1c39210	M04	MER001026	3.90E-87
ERIC1_2c07760	M04	MER001026	6.80E-87
ERIC1_1c24570	M09B	MER001417	2.20E-119
ERIC1_1c16780	M14C	MER001505	1.90E-78
ERIC1_1c31420	M15B	MER084164	5.90E-50
ERIC1_1c00860	M16B	MER142621	6.10E-90
ERIC1_1c00860	M16B	MER162087	2.60E-48
ERIC1_1c00990	M16B	MER071223	9.30E-40
ERIC1_1c00990	M16B	MER084990	4.40E-26
ERIC1_1c01000	M16B	MER013711	3.90E-85
ERIC1_1c01150	M19	MER013425	1.30E-51
ERIC1_1c10510	M20A	MER173790	3.80E-119
ERIC1_1c21340	M20A	MER001361	1.80E-89
ERIC1_1c25270	M20B	MER001421	6.70E-121

Table S1 continued

Gene ID	MEROPS family [1]	MEROPS entry	E-value*
ERIC1_2c04520	M20B	MER028941	2.40E-129
ERIC1_1c04100	M20D	MER005163	2.50E-10
ERIC1_1c07350	M20D	MER081890	4.50E-69
ERIC1_2c05580	M20D	MER005163	7.00E-91
ERIC1_4c00410	M20D	MER180918	3.10E-31
ERIC1_4c00420	M20D	MER180918	6.50E-26
ERIC1_1c26460	M22	MER145515	4.20E-78
ERIC1_1c29610	M22	MER038778	1.30E-26
ERIC1_1c29630	M22	MER001274	3.10E-74
ERIC1_1c11040	M23B	MER083041	6.30E-05
ERIC1_1c19070	M23B	MER145504	1.30E-05
ERIC1_1c25750	M23B	MER019259	1.20E-29
ERIC1_1c26840	M23B	MER158191	1.50E-14
ERIC1_1c27450	M23B	MER116068	5.90E-50
ERIC1_1c32440	M23B	MER145504	5.40E-08
ERIC1_1c32440	M23B	MER145504	5.50E-05
ERIC1_1c36830	M23B	MER088463	6.30E-14
ERIC1_2c07120	M23B	MER021826	4.20E-61
ERIC1_3c00550	M23B	MER083041	4.60E-06
ERIC1_1c16700	M24A	MER001243	6.20E-58
ERIC1_1c23010	M24A	MER001243	2.90E-62
ERIC1_1c01380	M24B	MER004931	2.00E-54
ERIC1_2c03980	M24B	MER004931	4.40E-67
ERIC1_2c06360	M24B	MER004931	4.30E-16
ERIC1_1c07010	M29	MER001285	1.10E-12
ERIC1_1c31500	M29	MER001287	1.70E-134
ERIC1_1c14980	M32	MER001186	3.00E-106
ERIC1_1c21750	M34	MER001345	8.40E-09
ERIC1_1c29960	M34	MER001345	9.70E-12
ERIC1_1c36220	M34	MER001345	8.80E-12
ERIC1_1c10560	M38	MER005767	1.00E-24
ERIC1_1c10560	M38	MER005767	8.00E-14
ERIC1_1c18770	M38	MER033184	8.00E-37
ERIC1_2c01690	M38	MER037714	2.20E-27
ERIC1_1c23810	M41	MER002602	3.50E-88
ERIC1_2c01720	M41	MER005496	7.60E-29
ERIC1_1c07850	M42	MER022215	1.90E-09
ERIC1_1c00710	M50B	MER004469	1.00E-13
ERIC1_1c00710	M50B	MER004480	3.40E-13
ERIC1_1c32730	M50B	MER038874	1.40E-08
ERIC1_1c36840	M50B	MER002454	9.30E-40
ERIC1_4c00400	M50B	MER004466	1.20E-05
ERIC1_1c04930	M56	MER014140	1.50E-05
ERIC1_1c29890	M60	MER042489	1.20E-07
ERIC1_1c35200	M60	MER042489	2.70E-36

Table S1 continued

Gene ID	MEROPS family [1]	MEROPS entry	E-value*
ERIC1_1c35200	M60	MER042489	4.60E-31
ERIC1_1c35210	M60	MER042489	6.90E-178
ERIC1_1c35220	M60	MER042489	1.10E-18
ERIC1_1c37500	M60	MER042489	2.20E-170
ERIC1_1c37500	M60	MER042489	6.90E-35
ERIC1_1c37520	M60	MER042489	4.00E-36
ERIC1_1c10380	M78	MER144929	1.60E-10
ERIC1_1c17570	M78	MER144929	4.10E-09
ERIC1_1c03250	S01B	MER102301	4.90E-43
ERIC1_1c33060	S01B	MER079071	2.30E-68
ERIC1_1c25680	S01X	MER038350	2.00E-52
ERIC1_1c05410	S08A	MER020842	1.50E-99
ERIC1_1c11210	S08A	MER166197	2.50E-99
ERIC1_1c19210	S08A	MER025143	9.30E-40
ERIC1_1c21520	S08A	MER020842	6.80E-87
ERIC1_1c26800	S08A	MER166197	1.70E-65
ERIC1_1c28090	S08A	MER081072	1.50E-65
ERIC1_1c28850	S08A	MER168175	2.00E-28
ERIC1_1c30570	S08A	MER000310	2.30E-51
ERIC1_1c30580	S08A	MER055152	9.60E-10
ERIC1_1c33350	S08A	MER090388	8.00E-57
ERIC1_2c05040	S08A	MER024807	6.40E-26
ERIC1_1c00030	S08X	MER082970	3.10E-39
ERIC1_1c13520	S08X	MER082970	3.30E-25
ERIC1_1c39150	S08X	MER082970	8.00E-41
ERIC1_1c12960	S09C	MER080940	2.90E-05
ERIC1_2c06050	S09C	MER074338	5.90E-18
ERIC1_1c09950	S09X	MER030913	2.80E-58
ERIC1_1c10430	S09X	MER031565	1.90E-06
ERIC1_1c19980	S09X	MER030913	2.60E-33
ERIC1_1c25010	S09X	MER030913	5.40E-23
ERIC1_1c05560	S11	MER137663	1.30E-47
ERIC1_1c26220	S11	MER028985	4.00E-89
ERIC1_2c00510	S11	MER040501	8.90E-123
ERIC1_2c00770	S11	MER137663	2.60E-105
ERIC1_1c02000	S12	MER065584	1.00E-25
ERIC1_1c04210	S12	MER028999	4.60E-68
ERIC1_1c21650	S12	MER041576	4.30E-44
ERIC1_1c00950	S14	MER020357	1.10E-59
ERIC1_1c08120	S14	MER085009	8.40E-62
ERIC1_1c12100	S14	MER085009	5.20E-17
ERIC1_1c12110	S14	MER085009	5.40E-39
ERIC1_1c28670	S14	MER125203	2.30E-87
ERIC1_1c33770	S14	MER085009	1.20E-33
ERIC1_1c35840	S14	MER085009	2.80E-60

Table S1 continued

Gene ID	MEROPS family [1]	MEROPS entry	E-value*
ERIC1_1c36400	S14	MER125203	4.30E-80
ERIC1_1c00150	S16	MER155619	4.10E-91
ERIC1_1c23530	S16	MER170752	6.40E-98
ERIC1_1c36450	S16	MER058049	1.70E-83
ERIC1_3c00660	S16	MER048375	2.50E-21
ERIC1_1c09510	S24	MER029010	3.90E-08
ERIC1_1c13670	S24	MER029010	9.70E-46
ERIC1_1c14910	S24	MER119211	2.00E-18
ERIC1_1c30680	S24	MER140304	7.60E-14
ERIC1_1c33440	S24	MER140304	6.50E-45
ERIC1_1c34850	S24	MER117873	5.20E-17
ERIC1_1c39370	S24	MER123765	3.10E-06
ERIC1_3c00900	S24	MER022307	3.90E-13
ERIC1_1c32390	S26A	MER055807	1.10E-42
ERIC1_2c03250	S26A	MER028421	1.50E-27
ERIC1_3c00460	S26A	MER028421	2.10E-37
ERIC1_1c02720	S33	MER031617	3.60E-11
ERIC1_2c03310	S33	MER044641	1.20E-11
ERIC1_2c03310	S33	MER045883	2.70E-07
ERIC1_2c06700	S33	MER036066	3.20E-09
ERIC1_1c27460	S41A	MER105195	7.10E-93
ERIC1_2c02580	S41A	MER123543	6.40E-12
ERIC1_2c04280	S55	MER003459	4.70E-92
ERIC1_1c31400	S58	MER164925	4.90E-26
ERIC1_1c31410	S58	MER164925	3.50E-21
ERIC1_1c29680	S66	MER025441	2.40E-17
ERIC1_1c00240	T01B	MER001626	4.80E-60
ERIC1_1c27360	T05	MER011829	6.30E-41
ERIC1_1c10740	U04	MER001293	2.20E-26
ERIC1_1c37420	U32	MER117225	6.50E-45
ERIC1_1c37430	U32	MER141870	2.40E-110
ERIC1_1c06390	U35	MER120958	2.50E-25
ERIC1_1c24100	U57	MER120195	7.10E-57
ERIC1_1c00490	U68	MER123660	6.30E-60
ERIC1_3c00420	U68	MER187143	5.00E-05

*E values of e-04 or less have were considered as significant

Reference

1. Rawlings ND, Morton FR (2008) The MEROPS batch Blast: a tool to detect peptidases and their non-peptidase homologues in a genome. Biochimie 90: 243-259.
