# Supplementary material for: How to Kill the Honey Bee Larva: Genomic Potential and Virulence Mechanisms of Paenibacillus larvae
Source: PLoS One. 2014 Mar 5;9(3):e90914. doi: 10.1371/journal.pone.0090914 (PMC3944939; doi:10.1371/journal.pone.0090914)
Supplement: Table S2 — Peptidases identified and classified in the genome of P. larvae strain DSM 25430. (PDF) [file pone.0090914.s003.pdf]

Table S2. Peptidases identified and classified in the genome of *P. larvae* strain DSM 25430.

Gene ID	MEROPS family [1]	MEROPS entry	E-value*
ERIC2_c24460	A08	MER070683	2.00E-12
ERIC2_c25740	A25	MER166302	7.70E-86
ERIC2_c02380	C26	MER066434	2.30E-08
ERIC2_c07940	C26	MER061258	1.40E-61
ERIC2_c24400	C26	MER146598	3.10E-89
ERIC2_c39480	C26	MER134379	3.90E-66
ERIC2_c06210	C40	MER003807	6.00E-18
ERIC2_c07300	C40	MER002450	4.50E-33
ERIC2_c08860	C40	MER003807	1.10E-44
ERIC2_c34370	C40	MER003805	7.20E-28
ERIC2_c38180	C40	MER003805	1.40E-27
ERIC2_c07830	C44	MER020221	4.90E-28
ERIC2_c08270	C44	MER004101	2.20E-64
ERIC2_c18310	C44	MER033254	1.40E-20
ERIC2_c28690	C44	MER033254	1.20E-16
ERIC2_c29880	C44	MER033254	8.50E-20
ERIC2_c37770	C44	MER003327	1.60E-60
ERIC2_c23100	C56	MER002455	3.90E-32
ERIC2_c41060	C56	MER031432	2.70E-11
ERIC2_c29130	C82	MER076166	2.20E-08
ERIC2_c31230	C82	MER076166	5.70E-05
ERIC2_c39100	M01	MER055808	1.90E-29
ERIC2_c11660	M03B	MER001163	6.70E-68
ERIC2_c30240	M03B	MER084913	1.10E-63
ERIC2_c17790	M04	MER001026	5.10E-104
ERIC2_c23330	M04	MER001026	5.20E-87
ERIC2_c25850	M04	MER001026	5.20E-87
ERIC2_c27330	M06	MER001164	7.00E-127
ERIC2_c40370	M09B	MER001417	4.40E-120
ERIC2_c11410	M14C	MER001505	1.90E-78
ERIC2_c36940	M15B	MER084164	1.30E-49
ERIC2_c21010	M16B	MER013711	2.00E-84
ERIC2_c21020	M16B	MER084990	1.00E-25
ERIC2_c21020	M16B	MER071223	9.30E-40
ERIC2_c21140	M16B	MER162087	2.00E-48
ERIC2_c21140	M16B	MER142621	6.10E-90
ERIC2_c20860	M19	MER013425	1.30E-51
ERIC2_c05420	M20A	MER001361	1.80E-89
ERIC2_c24360	M20A	MER173790	3.80E-119
ERIC2_c01850	M20B	MER001421	6.70E-121
ERIC2_c12590	M20B	MER028941	2.40E-129
ERIC2_c15490	M20D	MER081890	2.00E-67

Table S2 continued

Gene ID	MEROPS family [1]	MEROPS entry	E-value*
ERIC2_c18240	M20D	MER005163	2.50E-10
ERIC2_c22920	M20D	MER180918	3.10E-31
ERIC2_c22921	M20D	MER180918	6.50E-26
ERIC2_c00360	M22	MER145515	4.80E-77
ERIC2_c03500	M22	MER038778	1.30E-26
ERIC2_c03520	M22	MER001274	3.10E-74
ERIC2_c00710	M23B	MER158191	1.10E-14
ERIC2_c01320	M23B	MER116068	5.90E-50
ERIC2_c01580	M23B	MER019259	1.20E-29
ERIC2_c22020	M23B	MER083041	4.60E-06
ERIC2_c24890	M23B	MER083041	8.60E-05
ERIC2_c28230	M23B	MER088463	6.30E-14
ERIC2_c30340	M23B	MER021826	4.20E-61
ERIC2_c35810	M23B	MER145504	5.40E-08
ERIC2_c35810	M23B	MER166256	4.50E-06
ERIC2_c11500	M24A	MER001243	6.20E-58
ERIC2_c38280	M24A	MER001243	1.90E-61
ERIC2_c20680	M24B	MER004931	2.00E-54
ERIC2_c31110	M24B	MER004931	9.30E-17
ERIC2_c33050	M24B	MER004931	4.40E-67
ERIC2_c15870	M29	MER001285	1.10E-12
ERIC2_c36840	M29	MER001287	2.20E-134
ERIC2_c27270	M32	MER001186	2.30E-106
ERIC2_c05310	M34	MER001345	2.00E-12
ERIC2_c28760	M34	MER001345	6.00E-08
ERIC2_c07350	M38	MER033184	8.00E-37
ERIC2_c14070	M38	MER037714	2.20E-27
ERIC2_c24410	M38	MER005767	1.00E-24
ERIC2_c24410	M38	MER005767	8.00E-14
ERIC2_c14100	M41	MER005496	1.10E-28
ERIC2_c39570	M41	MER002602	3.50E-88
ERIC2_c21290	M50B	MER004469	1.00E-13
ERIC2_c21290	M50B	MER004480	3.40E-13
ERIC2_c22910	M50B	MER004466	8.90E-06
ERIC2_c28220	M50B	MER002454	7.00E-38
ERIC2_c35500	M50B	MER038874	1.40E-08
ERIC2_c08430	M78	MER144929	2.50E-11
ERIC2_c24210	M78	MER144929	1.60E-10
ERIC2_c01340	S01B	MER087818	8.30E-05
ERIC2_c35160	S01B	MER079071	2.30E-68
ERIC2_c01640	S01X	MER038350	1.20E-52
ERIC2_c00670	S08A	MER166197	3.00E-65
ERIC2_c04380	S08A	MER000310	2.30E-51
ERIC2_c04390	S08A	MER138281	8.90E-06
ERIC2_c09000	S08A	MER025143	9.30E-40

Table S2 continued

Gene ID	MEROPS family [1]	MEROPS entry	E-value*
ERIC2_c34730	S08A	MER090388	8.00E-57
ERIC2_c39030	S08A	MER081072	1.50E-65
ERIC2_c29670	S09C	MER080940	2.90E-05
ERIC2_c31490	S09C	MER074338	5.90E-18
ERIC2_c07680	S09X	MER030913	2.60E-33
ERIC2_c23880	S09X	MER030913	2.80E-58
ERIC2_c24270	S09X	MER031565	1.90E-06
ERIC2_c40780	S09X	MER030913	4.70E-23
ERIC2_c00130	S11	MER028985	4.00E-89
ERIC2_c12800	S11	MER040501	2.30E-123
ERIC2_c13110	S11	MER137663	2.60E-105
ERIC2_c05200	S12	MER041576	1.10E-29
ERIC2_c18130	S12	MER028999	4.60E-68
ERIC2_c20000	S12	MER065584	1.00E-25
ERIC2_c02560	S14	MER125203	2.30E-87
ERIC2_c21060	S14	MER020357	1.10E-59
ERIC2_c28620	S14	MER125203	1.50E-80
ERIC2_c21870	S16	MER155619	1.60E-94
ERIC2_c22120	S16	MER048375	7.90E-21
ERIC2_c28570	S16	MER058049	1.70E-83
ERIC2_c28580	S16	MER164590	1.70E-79
ERIC2_c28580	S16	MER155619	1.60E-07
ERIC2_c38790	S16	MER170752	6.40E-98
ERIC2_c04500	S24	MER140304	7.60E-14
ERIC2_c25960	S24	MER029010	9.70E-46
ERIC2_c27180	S24	MER119211	2.00E-18
ERIC2_c21930	S26A	MER028421	7.00E-38
ERIC2_c31810	S26A	MER028421	1.50E-27
ERIC2_c35880	S26A	MER055807	1.10E-42
ERIC2_c18550	S33	MER036050	8.50E-21
ERIC2_c30770	S33	MER036066	5.30E-10
ERIC2_c31750	S33	MER044641	1.20E-11
ERIC2_c31750	S33	MER045883	2.70E-07
ERIC2_c01330	S41A	MER105195	2.10E-92
ERIC2_c32750	S55	MER003459	4.70E-92
ERIC2_c36941	S58	MER164925	3.50E-21
ERIC2_c36950	S58	MER100239	8.40E-05
ERIC2_c36950	S58	MER164925	4.90E-26
ERIC2_c03570	S66	MER142981	4.20E-19
ERIC2_c21760	T01B	MER001626	1.40E-59
ERIC2_c01230	T05	MER011829	4.80E-41
ERIC2_c24590	U04	MER001293	2.20E-26
ERIC2_c05010	U32	MER019303	7.20E-59
ERIC2_c05010	U32	MER019303	1.80E-09
ERIC2_c27620	U32	MER117225	1.20E-111

Table S2 continued

**Gene ID**	**MEROPS family [1]**	**MEROPS entry**	**E-value***
ERIC2_c27630	U32	MER117225	6.50E-45
ERIC2_c39860	U57	MER120195	7.10E-57
ERIC2_c21510	U68	MER123660	6.30E-60
ERIC2_c21890	U68	MER187143	5.00E-05

*E values of e-04 or less were considered as significant

Reference

1. Rawlings ND, Morton FR (2008) The MEROPS batch Blast: a tool to detect peptidases and their non-peptidase homologues in a genome. Biochimie 90: 243-259.
