# Supplementary material for: How to Kill the Honey Bee Larva: Genomic Potential and Virulence Mechanisms of Paenibacillus larvae
Source: PLoS One. 2014 Mar 5;9(3):e90914. doi: 10.1371/journal.pone.0090914 (PMC3944939; doi:10.1371/journal.pone.0090914)
Supplement: Table S3 — P. larvae strains used in this study. (PDF) [file pone.0090914.s004.pdf]

Table S3. *P. larvae* strains used in this study.

Strain	Source	ERIC genotype
ATCC 9545	ATCC	I
DSM 25719	Honey (dis. col.)	I
02-075	Honey (dis. col.)	I
02-081	Honey (dis. col.)	I
02-179	Honey (dis. col.)	I
02-250	Honey (dis. col.)	I
03-119	Honey (dis. col.)	I
03-122	Honey (dis. col.)	I
03-189	Honey (dis. col.)	I
00-1163	Honey (dis. col.)	I I
01-1714	Honey (dis. col.)	I I
03-016	Honey (dis. col.)	I I
03-195	Honey (dis. col.)	I I
03-199	Honey (dis. col.)	I I
03-200	Honey (dis. col.)	I I
03-522	Honey (dis. col.)	I I
03-478	Honey (dis. col.)	I I
03-518	Honey (dis. col.)	I I
03-525	Honey (dis. col.)	I I
DSM 25430	Honey (dis. col.)	I I

(dis. col.), AFB-diseased colony.
