# Supplementary material for: How to Kill the Honey Bee Larva: Genomic Potential and Virulence Mechanisms of Paenibacillus larvae
Source: PLoS One. 2014 Mar 5;9(3):e90914. doi: 10.1371/journal.pone.0090914 (PMC3944939; doi:10.1371/journal.pone.0090914)
Supplement: Table S4 — CRISPR analysis of the P. larvae strain DSM 25719 genome. (PDF) [file pone.0090914.s005.pdf]

Table S4. CRISPR analysis of the *P. larvae* strain DSM 25719 genome.

Contig	CRISPR	DR consensus	Spacer	Start	Stop	CRISPR length	DR length	Number of spacers
1c	Crispr_1	TTTGCAATTCAACTATTCGCGTAGGATGCGAC	ATAGCCGCGCTATATGGCGGTCGGCTTAATTG	590070	590133	364	32	5
		ATTTCAATCCACGCATCCGCGTAGGATGCGAC	ATTGAAAACCAAAATAACCTTTCGGAAATTATCC	590134	590199			
		ATTTCAATCCACGCATCCGCGTAGGATGCGAC	GGGAATAGCAATCAATTCGGTTTGTATTTTGATGATG	590200	590268			
		ATTTCAATCCACGCATCCGCGTAGGATGCGAC	ATTTACATCTGCTTCAGTTGCATATTTGTAATTGTT	590269	590336			
		ATTTCAATCCACGCATCCGCGTAGGATGCGAC	AGGAATTGTTTCCTGAGGGTGTTTCAGATGATGT	590337	590402			
		ATTTCAATCCACGCATCCGCGTAGGATGCGAC		590403	590434			
	Crispr_2	ATTTCAATCCACGCATCCACATGGGATGCGAC	CTTTAGCGTTGTTCGAAACTGCAATTACTTCATG	592753	592818	368	32	5
		ATTTCAATCCACGCATCCGCGTAGGATGCGAC	GCGTGCGGACGGCCGCCACATAATCGGGGTTTTT	592819	592884			
		ATTTCAATCCACGCATCCGCGTAGGATGCGAC	ATTCGCCTGTCGCCATATGACACTGGCCACAGAACG	592885	592952			
		ATTTCAATCCACGCATCCGCGTAGGATGCGAC	CATATCGTGAGTCAGGCGACTTACGATTCATACCGT	592953	593020			
		ATTTCAATCCACGTATCCGCGTAGGATGCGAC	ACCCTTCGGCTTCTGCAACAGCAGATACCCGGTCATA	593021	593089			
		ATTTCAATCCACGCATCCGCGTAGGATGCGAC		593090	593121			
	Possible Crispr_3	TGTTACTTTGCTGTTACTTTACT	GTTACCCCAAACATTTTTGATAGGCTCTAATCCCTTGATATATAAGGGTTTAAGG	848780	848857	100	23	1
		TGTTACTTTGCTGTTACCCCAAA		848858	848880			
	Possible Crispr_4	TGTTACTTTGCTGTTACTTTACT	GTTACCCCAAACATTTTTGATAGGCTCTAATCCCTTGATATATAAGGGTTTAAGG	1166305	1166382	100	32	1
		TGTTACTTTGCTGTTACCCCAAA		1166383	1166405			
	Possible Crispr_5	TTTTTTCATTAAATCTTTTTCGG	TAACTTTCCCCACCAGCCCTTTTTCGGCAAA	3295955	3296008	76	23	1
		TTTTTTCATCAAATCTTTTTCGG		3296009	3296031			
2c	Crispr_1	GTTTGATAGTAACACGAGGTGTATTGAAAC	TGGTGTGGGTGATTCCGTGTCCGTCCACCATTTGCC	514470	514535	228	30	3
		GTTTGATAGTAACACGAGGTGTATTGAAAC	GGAAAAGTATCAGGCAGACGAATACCGAATCATTCC	514536	514601			
		GTTTGATAGTAACACGAGGTGTATTGAAAC	AGTTCTTCCATTTCCCAATACTCTTCCGTTTCCGTAT	514602	514668			
		GTTTGAAAGTAACACAAGGTATATTGAACT		514669	514698			
	Crispr_2	GTTTGACAGTAACACATGGTGTATTGAAACT	ATTGCCCGCCGCGTGGGCGTCGTACCTGCCCTT	524249	524312	292	31	4
		GTTTGACAGTAACACATGGTGTATTGAAACT	GCAGTAGCGACAGTGTATTCAGCAGTTATAAGTAG	524313	524378			
		GTTTGACAGTAACACATGGTGTATTGAAACT	TCACACAGCCCTCCTCAAATTATCCGCCCTTTGGC	524379	524444			
		GTTTGACAGTAACACATGGTGTATTGAAACT	GGAGATGAAAGCACTTTGTGAACAAAAAGAATTGG	524445	524510			
		GTTTGACAGTAACACAAGGTGTATTGAAACG		524511	524541			
