# Supplementary material for: How to Kill the Honey Bee Larva: Genomic Potential and Virulence Mechanisms of Paenibacillus larvae
Source: PLoS One. 2014 Mar 5;9(3):e90914. doi: 10.1371/journal.pone.0090914 (PMC3944939; doi:10.1371/journal.pone.0090914)
Supplement: Table S5 — CRISPR analysis of the P. larvae strain DSM 25430 genome. (PDF) [file pone.0090914.s006.pdf]

Table S5. CRISPR analysis of the *P. larvae* strain DSM 25430 genome.

CRISPR	Direct repeat consensus	Spacer	Start	Stop	CRISPR length	Direct repeat length	No. of spacers
Crispr_1	GTCGCATCCTACGCGGATGCGTGGATTGAAATT	TGTTTCGATGCTATATTTATACGCATCAATA	1647332	1647395	566	33	8
	GTCGCATCCTACGCGGATGCGTGGATTGAAATA	AGGGAGGGGATTTTCAGACTCAGTTTAGACTATAC	1647396	1647463			
	GTCGCATCCTACGCGGATGCGTGGATTGAAATA	TTACAGGGGCAGGGAGGTACAGAAGATAGGAGGTAC	1647464	1647532			
	GTCGCATCCTACGCGGATGCGTGGATTGAAATA	CTAGAGCAATGAGCATTAACGGGATTCCAATCA	1647533	1647598			
	GTCGCATCCTACGCGGATGCGTGGATTGAAATA	AGGGAGGGGATTTTCAGACTCAGTTTAGACTATAC	1647599	1647666			
	GTCGCATCCTACGCGGATGCGTGGATTGAAATA	TTACAGGGGCAGGGAGGTACAGAAGATAGGAGGTAC	1647667	1647735			
	GTCGCATCCTACGCGGATGCGTGGATTGAAATA	CTAGAGCAATGAGCATTAACGGGATTCCAATCA	1647736	1647801			
	GTCGCATCCTACGCGGATGCGTGGATTGAAATC	AATTAAGCCGACCGCCATATAGCGTGGCTAT	1647802	1647865			
	GTCGCATCCTACGCGAATAGTTGAATTGCAAAT		1647866	1647898			
