# Supplementary material for: How to Kill the Honey Bee Larva: Genomic Potential and Virulence Mechanisms of Paenibacillus larvae
Source: PLoS One. 2014 Mar 5;9(3):e90914. doi: 10.1371/journal.pone.0090914 (PMC3944939; doi:10.1371/journal.pone.0090914)
Supplement: Table S6 — Strain-specific regions identified in the genome of P. larvae strain DSM 25719. (PDF) [file pone.0090914.s007.pdf]

Table S6. Strain-specific regions identified in the genome of *P. larvae* strain DSM 25719.

Locus	ORFs		Number of CDS	Region position		Region length (kbp)	Features
**GI1**	ERIC1_1c00010	ERIC1_1c00140	14	881	12650	11.8	Toxin locus Plx1, hypothetical proteins
**GI2**	ERIC1_1c01240	ERIC1_1c01300	7	124303	130500	6.2	Toxin locus Plx5, hypothetical proteins
**GI3**	ERIC1_1c01940	ERIC1_1c02140	21	200595	229098	28.5	Insertion elements, PKS/NRPS cluster
**GI4**	ERIC1_1c03210	ERIC1_1c03360	15	339552	352751	13.2	Putative serine protease HtrA, transcriptional regulator, hypothetical proteins
**GI5**	ERIC1_1c03660	ERIC1_1c04000	35	399709	430598	30.9	Type-2 restriction enzyme BsuBI, integrase-recombinase protein, hypothetical proteins, insertion elements, putative O-methyltransferase, phenolic acid decarboxylase PadC, negative transcription regulator PadR, methylinsertion elementsocitrate lyase PrpB, 2-methylcitrate dehydratase PrpD, 2-methylcitrate synthase MmgD, heme-degrading monooxygenase (iron transport system insertion elementsdCDEFG), sortase B, bifunctional phosphonoacetaldehydehydrolase/a minoethylphosphonate transaminase
**GI6**	ERIC1_1c07610	ERIC1_1c07770	17	806692	816029	9.3	Oxidoreductase, glycosyltransferase, replicative DNA helicase, transcriptional regulator, insertion elements, hypothetical proteins
**GI7**	ERIC1_1c09620	ERIC1_1c09730	12	934451	940913	6.4	Insertion elements, hypothetical proteins, ERIC1_1c09720 toxin-like protein
**GI8**	ERIC1_1c13750	ERIC1_1c13850	11	1323964	1332635	8.7	Insertion elements, hypothetical proteins, toxin locus Plx3
**GI9**	ERIC1_1c15090	ERIC1_1c15270	19	1431556	1446376	14.9	ERIC1_1c15200 ricin-type beta-trefoil lectin domain protein, insertion elements, hypothetical proteins
**GI10**	ERIC1_1c19060	ERIC1_1c19180	13	1789752	1809398	19.7	SMC domain protein, type I restriction-modification system,lantibiotic modifying enzyme
**GI11**	ERIC1_1c21120	ERIC1_1c21210		2001170	2007677	6.5	Resolvase, putative bacteriocin, hypothetical proteins
**GI12**	ERIC1_1c21410	ERIC1_1c21560	16	2025054	2035702	10.6	Insertion elements, hypothetical proteins, subtilinsertion elementsin-like serine protease, bacitracin export ATP-binding protein BceA, efflux ABC transporter, permease protein
**GI13**	ERIC1_1c21700	ERIC1_1c21880	19	2048032	2063431	15.4	Insertion elements, hypothetical proteins, toxin locus Tx6
**GI14**	ERIC1_1c22330	ERIC1_1c22400	8	2111904	2119733	7.8	Oxidoreductase, PTS-dependent dihydroxyacetone kinase,dihydro xyacetone-binding subunit DhaK, glycerol dehydrogenase DhaD, transcriptional regulator, AraC family, chloramphenicol O-acetyltransferase CatB, hypothetical proteins
**GI15**	ERIC1_1c25490	ERIC1_1c25570	9	2419728	2430230	10.5	ATP-dependent dethiobiotin ligase BioD, adenosylmethionine-8-amino-7-oxononanoateaminotransferase BioA, serine/threonine exchanger SteT, putative 4-methyl-5(B-hydroxyethyl)-thiazolem onophosphate biosynthesinsertion elements enzyme
**GI16**	ERIC1_1c29800	ERIC1_1c29900	11	2835914	2844681	8.8	Integrase, insertion elements, hypothetical proteins, serine alkaline protease-like protein, N-acetylmuramoyl-L-alanine amidase

Table S6 continued

Locus	ORFs		Number of CDS	Region position		Region length (kbp)	Features
**GI17**	ERIC1_1c29960	ERIC1_1c30070	14	2854019	2859218	5.2	Hypothetical proteins, toxin locus Plx4
**GI18**	ERIC1_2c00010	ERIC1_2c00320	33	179	18564	18.4	Insertion elements, hypothetical proteins
**GI19**	ERIC1_2c02250	ERIC1_2c02370	13	199255	219958	20.8	RHS repeat-associated core domain-containing protein, hypothetical proteins, insertion elements,secreted proteins, cell surface protein
**GI20**	ERIC1_2c02510	ERIC1_2c02580	8	233196	240873	7.7	Flavohemoprotein, putative amidinotransferase, hypothetical proteins, insertion elements
**GI21**	ERIC1_2c04540	ERIC1_2c05970	143	419125	542360	123.2	Insertion elements, hypothetical proteins, putative cation-transporting ATPase, Carbohydrate metabolinsertion elementsm uxuABC, PTS system, glycosyltransferases, aminotransferases, transcriptional regulators, toxin locus Tx7, subtilinsertion elementsin-like serine proteases, ferrous iron transport proteins, amino acid permease, CRinsertion elementsPR-associated protein Cas,
**GI22**	ERIC1_4c00710	ERIC1_4c00800	10	71976	77047	5.1	Hypothetical proteins, ABC transporter-like protein, N-acetylmuramoyl-L-alanine amidase
**GI23**	ERIC1_7c00010	ERIC1_7c00090	9	271	7600	7.3	Hypothetical proteins, N-acetylmuramoyl-L-alanine amidase, Ricin-type beta-trefoil lectin domain-like protein
