# Supplementary material for: How to Kill the Honey Bee Larva: Genomic Potential and Virulence Mechanisms of Paenibacillus larvae
Source: PLoS One. 2014 Mar 5;9(3):e90914. doi: 10.1371/journal.pone.0090914 (PMC3944939; doi:10.1371/journal.pone.0090914)
Supplement: Table S7 — Strain-specific regions identified in the genome of P. larvae strain DSM 25430. (PDF) [file pone.0090914.s008.pdf]

Table S7. Strain-specific regions identified in the genome of *P. larvae* strain DSM 25430.

Locus	ORFs		Number of CDS	Region position		Region lenght (Kbp)	Features
**GI1**	ERIC2_c18480	ERIC2_c18620	15	1818437	1829521	11.1	Insertion elements, hypothetical proteins, amidinotransferase, alpha/beta hydrolase, monogalactosyldiacylglycerol synthase-like protein
**GI2**	ERIC2_c18730	ERIC2_c18760	4	1839400	1865129	25.7	PKS/NRPS cluster
**GI3**	ERIC2_c19270	ERIC2_c19330	7	1925490	1932807	7.3	Insertion elements, hypothetical proteins, bacitracin export ATP-binding protein BceA
